# Hybrid Molecular and Functional Micro-CT Imaging Reveals Increased Myocardial Apoptosis Preceding Cardiac Failure in Progeroid *Ercc1* Mice

**DOI:** 10.1007/s11307-024-01902-4

**Published:** 2024-03-18

**Authors:** Bibi S. van Thiel, Martine de Boer, Yanto Ridwan, Marion G. J. de Kleijnen, Nicole van Vliet, Janette van der Linden, Isa de Beer, Paula M. van Heijningen, Wilbert P. Vermeij, Jan H. J. Hoeijmakers, A. H. Jan Danser, Roland Kanaar, Dirk J. Duncker, Ingrid van der Pluijm, Jeroen Essers

**Affiliations:** 1 Department of Molecular Genetics, Erasmus MC Cancer Institute, Erasmus University Medical Center, Rotterdam, The Netherlands; 2 Department of Vascular Surgery, Erasmus MC Cardiovascular Institute, Erasmus University Medical Center, Room 702A, Wytemaweg 80, 3015 CN Rotterdam, The Netherlands; 3 Division of Vascular Medicine and Pharmacology, Department of Internal Medicine, Erasmus University Medical Center, Rotterdam, The Netherlands; 4 Division of Experimental Cardiology, Department of Cardiology, Erasmus MC Cardiovascular Institute, Erasmus University Medical Center, Rotterdam, The Netherlands; 5 Department of Radiotherapy, Erasmus University Medical Center, Room 702A, Wytemaweg 80, 3015 CN Rotterdam, The Netherlands; 6 Princess Máxima Center for Pediatric Oncology, Utrecht, The Netherlands; 7 Oncode Institute, Utrecht, The Netherlands; 8 Institute for Genome Stability in Aging and Disease, Cologne Excellence Cluster for Cellular Stress Responses in Aging-Associated Diseases (CECAD), University of Cologne, Cologne, Germany

**Keywords:** Molecular imaging, Aging, Heart failure, DNA repair, Ercc1, FMT, CT

## Abstract

**Purpose:**

In this study, we explored the role of apoptosis as a potential biomarker for cardiac failure using functional micro-CT and fluorescence molecular tomography (FMT) imaging techniques in *Ercc1* mutant mice. Ercc1 is involved in multiple DNA repair pathways, and its mutations contribute to accelerated aging phenotypes in both humans and mice, due to the accumulation of DNA lesions that impair vital DNA functions. We previously found that systemic mutations and cardiomyocyte-restricted deletion of *Ercc1* in mice results in left ventricular (LV) dysfunction at older age.

**Procedures and Results:**

Here we report that combined functional micro-CT and FMT imaging allowed us to detect apoptosis in systemic *Ercc1* mutant mice prior to the development of overt LV dysfunction, suggesting its potential as an early indicator and contributing factor of cardiac impairment. The detection of apoptosis *in vivo* was feasible as early as 12 weeks of age, even when global LV function appeared normal, underscoring the potential of apoptosis as an early predictor of LV dysfunction, which subsequently manifested at 24 weeks.

**Conclusions:**

This study highlights the utility of combined functional micro-CT and FMT imaging in assessing cardiac function and detecting apoptosis, providing valuable insights into the potential of apoptosis as an early biomarker for cardiac failure.

**Supplementary Information:**

The online version contains supplementary material available at 10.1007/s11307-024-01902-4.

## Introduction

Heart failure has become a global disease epidemic, particularly in the elderly [1, 2]. Notwithstanding major advances in treatment, the exact molecular mechanisms underlying the pathogenesis of heart failure remain incompletely understood, hampering a more mechanism-based approach to effective treatment and prevention. The accumulation of unrepaired DNA damage over time is one of the most important hallmarks of aging [3, 4] and is regarded as one of the driving forces of accelerated aging and age-related diseases, including heart failure [5–7].

A variety of these genetically altered mice with affected DNA repair pathways demonstrate a strict correlation between the severity of the DNA repair defect and the severity of aging pathology and lifespan, suggesting that the load of DNA damage directly relates to the rate of aging [8–10]. Several lines of evidence in experimental as well as clinical studies, support the notion that accumulation of DNA damage, secondary to oxidative stress, is involved in the development of age-related cardiovascular diseases (CVDs) [11]. The discovery by Gyenis et al. [12] of DNA damage-induced transcription stress, which is dependent on gene length and occurs in postmitotic tissues, holds significant importance as it establishes a compelling connection between DNA damage and the majority of the hallmark processes associated with systemic aging. Yet, the precise role of DNA damage and related aging on the manifestation of CVDs remains elusive and needs further exploration.

In the present study, we employed mice with systemic or cardiomyocyte-restricted deficient DNA repair due to a defect in the *Ercc1* gene. Systemic *Ercc1*^Δ/−^ mice contain one knockout allele of *Ercc1* and one protein truncating mutation, in which the last seven amino acids at the C-terminus of the Ercc1 protein are deleted [13]. In addition, we studied mice with cardiomyocyte-restricted *Ercc1* deficiency generated by the *Cre-loxP* system [14, 15]). Both systemic and cardiomyocyte-restricted *Ercc1* mutants are deficient in multiple DNA repair mechanisms including nucleotide excision repair, interstrand crosslink repair and homologous recombination [13, 16], and exhibit marked left ventricular (LV) dysfunction [14]. To investigate whether apoptosis is an early marker of cardiac remodeling and dysfunction, we studied these mutant mice at different time points, using hybrid micro-CT and FMT imaging with the Annexin-Vivo that binds to phosphatidylserine which is exposed on the outer leaflet of the cell membrane lipid bilayer during the early stages of apoptosis to enable non-invasive *in vivo* molecular imaging of myocardial apoptosis in relation to LV function.

The combination of micro-CT functional cardiovascular analysis and fluorescence molecular apoptosis imaging offers a powerful synergy in cardiovascular research. It provides the ability to simultaneously visualize and quantify functional parameters of the cardiovascular system with high-resolution imaging, while also detecting and assessing apoptosis at the molecular level.

## Methods

### Mouse Models

All animal procedures were performed in accordance with the Principles of Laboratory Animal Care and Guidelines approved by the Dutch Animal Ethical Committee in full accordance with European legislation. As required by Dutch law, formal permission to generate and use genetically modified animals was obtained from the responsible local and national authorities. A total of 85 mice, males and females, were used for this study. Animals were housed in individual ventilated cages under specific pathogen free conditions and maintained in a controlled environment (20–22 ˚C, 12 h light: 12 h dark cycle). They were given ad libitum access to food, maintained on either AIN93G synthetic pellets (Research Diet Services B.V.; gross energy content 4.9 kcal/g dry mass, digestible energy 3.97 kcal/g) or standard chow diet and water.

The generation of *Ercc1*^*Δ/−*^ mutants (carrying a genetic mutation in which one of the alleles is removed, “knock-out”, and the other allele has a C-terminally truncation, “delta”, leading to the production of a truncated version of the Ercc1 protein (*Ercc1*^*Δ/−*^)) and their wild-type *Ercc1*^+*/*+^ littermates (WT) has been described previously [13]. These mice, all in a F1 hybrid FVB/C57BL/6 J background, were studied at 6, 12 and 24 weeks of age. Typical unfavorable characteristics, such as blindness in the FVB background or deafness in the C57BL/6 J background, do not occur in this hybrid background. Mice with cardiomyocyte-restricted deletion of *Ercc1* (*αMHC-Ercc1*^*c/−*^) were generated using the *Cre-loxP* technology as previously described [14]. Mice harboring two conditional alleles of *Ercc1* were crossed with hemizygous mice expressing Cre-recombinase under the control of the α-myosin heavy chain (*αMHC-Cre*) promotor [17]*. αMHC-Ercc1*^*c/−*^ mice and their control littermates (Control) harboring the same F1 hybrid FVB/C57BL/6 J background, were studied at 8 and 16 weeks of age.

### *In Vivo* Micro-CT Imaging of LV Function

Mice were imaged with contrast enhanced Quantum FX Micro-computed Tomography (micro-CT) (Perkin Elmer Inc., Akron, Ohio, USA) for anatomical reference and to assess cardiac morphology and function [18].

Mice were anaesthetized (1.5–2.5% isoflurane, O_2_ 1 L/min) and depilated to minimize the interference of fur on the fluorescent signal during FMT imaging. Prior to micro-CT imaging (Perkin Elmer Inc.), mice were injected in the tail vein with 125 µL per 25 g body weight of the iodinated contrast agent eXIA160 (10 mg/ml, Binitio Biomedical Inc., Ottawa, Canada), positioned in the multimodal imaging cassette and restrained to prevent movement during imaging. Mice were scanned using intrinsic cardiac respiratory gating to reduce artifacts caused by breathing or cardiac motion. After micro-CT imaging, mice remained under anesthesia and the cassette was transferred to the FMT 2500 fluorescent tomography *in vivo* imaging system (Perkin Elmer Inc.). Indexes of LV myocardial mass (LVMM) and LV function (LV end-diastolic volume, LV end-systolic volume and LV stroke volume) were measured from the 3D micro-CT images using the software ANALYZE **®** 12.0 (AnalyzeDirect Inc., Overland Parks, KS, USA). Subsequently, stroke volume was calculated as LV-end-diastolic volume - LV end-systolic volume, while LV ejection fraction was calculated using this formula: ((LV end-diastolic volume-LV end-systolic volume)/LV end-diastolic volume)*100%.

### *In Vivo* FMT Imaging of Myocardial Apoptosis

The near-infrared fluorescence (NIRF) probe Annexin-Vivo750™ (Perkin Elmer Inc.) was used to image myocardial apoptosis. Therefore, the FMT 2500 fluorescence tomography *in vivo* imaging system (Perkin Elmer Inc.) was used. Two hours before imaging, mice were injected intravenously with Annexin-Vivo 750™ (according to manufacturer’s instructions). After finishing micro-CT imaging, the cassette was transferred to the FMT 2500 fluorescent tomography *in vivo* imaging system (Perkin Elmer Inc.). FMT imaging was performed using 750 and 770–800 nm excitation and emission wavelengths, respectively. The multimodal imaging cassette facilitates the co-registration of FMT and micro-CT data through fiducial landmarks. FMT and micro-CT data were merged using the TrueQuant software (Perkin Elmer Inc.) and *in vivo* cardiac fluorescence was quantified as the amount of pmol divided by heart weight (g).

### *Ex Vivo* Fluorescent Imaging of Excised Hearts

Immediately after *in vivo* micro-CT and FMT imaging, mice were euthanized using an overdose of inhalant anesthetic isoflurane. Hearts were excised, weighed, immersion fixed in formalin for 1 day and assessed for *ex vivo* tissue epifluorescence using the Odyssey® CLx imaging system (LI-COR® Biosciences, Lincoln, Nebraska, USA). *Ex vivo* cardiac fluorescence was quantified as counts per mm^2^.

### Immunohistochemical Analysis

After formalin fixation, the heart was transversally sectioned into two parts and stored overnight in 70% ethanol. Subsequently, hearts were embedded in paraffin, sectioned at 5 µm, and mounted on Superfrost Plus slides. Cross-sections of the whole heart were stained for TUNEL (terminal deoxynucleotidyl transferase-mediated dUTP nick end labelling, ApopTag In Situ Apoptosis Kit (Oncor)) to determine the amount of apoptotic cells using standard procedure. TUNEL staining was quantified by counting TUNEL positive (dark brown) cells in qualitatively suitable sections from the heart. This number was corrected for the size of the quantified area by dividing the total fragmentation count by the surface of the measured area (cells/mm^2^).

### Statistical Analysis

Data are expressed as mean ± SEM. Survival analysis was performed with the Mantel-Cox test using GraphPad Prism 10.1.0. Differences between groups were evaluated by two-way ANOVA, followed by Student–Newman–Keuls (SNK) post-hoc testing using SigmaPlot 11.0. *P* < 0.05 (two-tailed) was considered significant.

## Results

### Anatomical Features

Genetically modified *Ercc1*^*∆/−*^ mice are commonly used to study the effects of defective DNA repair mechanisms and the consequences of DNA damage accumulation. As previously described, *Ercc1*^*∆/−*^ mice showed reduced growth, declined body weight and a severely shortened lifespan (maximally 30 weeks, *p < 0.0001) compared to their WT littermates (Suppl. Figure 1 and Table 1) [10]. We compared this systemic deletion of the *Ercc1* gene with cardiomyocyte-restricted deletion of the *Ercc1* gene to distinguish and analyze *Ercc1*’s specific role in the heart, in order to provide insights into the role of DNA damage repair in cardiac function, cardiac aging and disease mechanism. *αMHC-Ercc1*^*c/−*^ mice displayed similar body and heart weight compared to their control littermates (Table 1), however, they displayed a similar severely shortened lifespan (maximally 24 weeks) as *Ercc1*^*∆/−*^ mice [14].

**Table 1:** Anatomical data of *Ercc1*^*∆/−*^ and their WT littermates at 6, 12 and 24 weeks of age, and *αMHC-Ercc1*^*c/−*^ and their control littermates at 8 and 16 weeks of age

		Systemic			
		WT		*Ercc1*^*Δ/−*^	
		n		n	
Body weight (g)	6 weeks	6	23.1 ± 1.5	6	15.5 ± 0.9 †
	12 weeks	8	29.0 ± 2.4 *	8	14.9 ± 0.6 †
	24 weeks	11	36.0 ± 2.4 *‡	22	13.3 ± 0.2 † §
Heart weight (mg)	6 weeks	6	134 ± 6	6	87 ± 3 †
	12 weeks	8	133 ± 7	8	75 ± 3 †
	24 weeks	11	148 ± 7	22	77 ± 1 † §
		Conditional			
		Control		*αMHC-Ercc1 *^*c/−*^	
		n		n	
Body weight (g)	8 weeks	6	22.0 ± 1.2	6	22.4 ± 1.3
	16 weeks	6	27.8 ± 3.3	6	25.6 ± 2.2
Heart weight (mg)	8 weeks	6	113 ± 4	6	113 ± 5
	16 weeks	6	136 ± 7 *	6	135 ± 8 *

Data presented as mean ± SEM

^*^p < 0.05 vs. corresponding 6 or 8 weeks; ‡*p* < 0.05 vs. corresponding 12 weeks †*p* < 0.05 vs. age-matched WT or Control; § *p* < 0.05 interaction genotype x age

### Quantitative *In Vivo* Micro-CT Assessment of LV Myocardial Mass

**Fig. 1.**
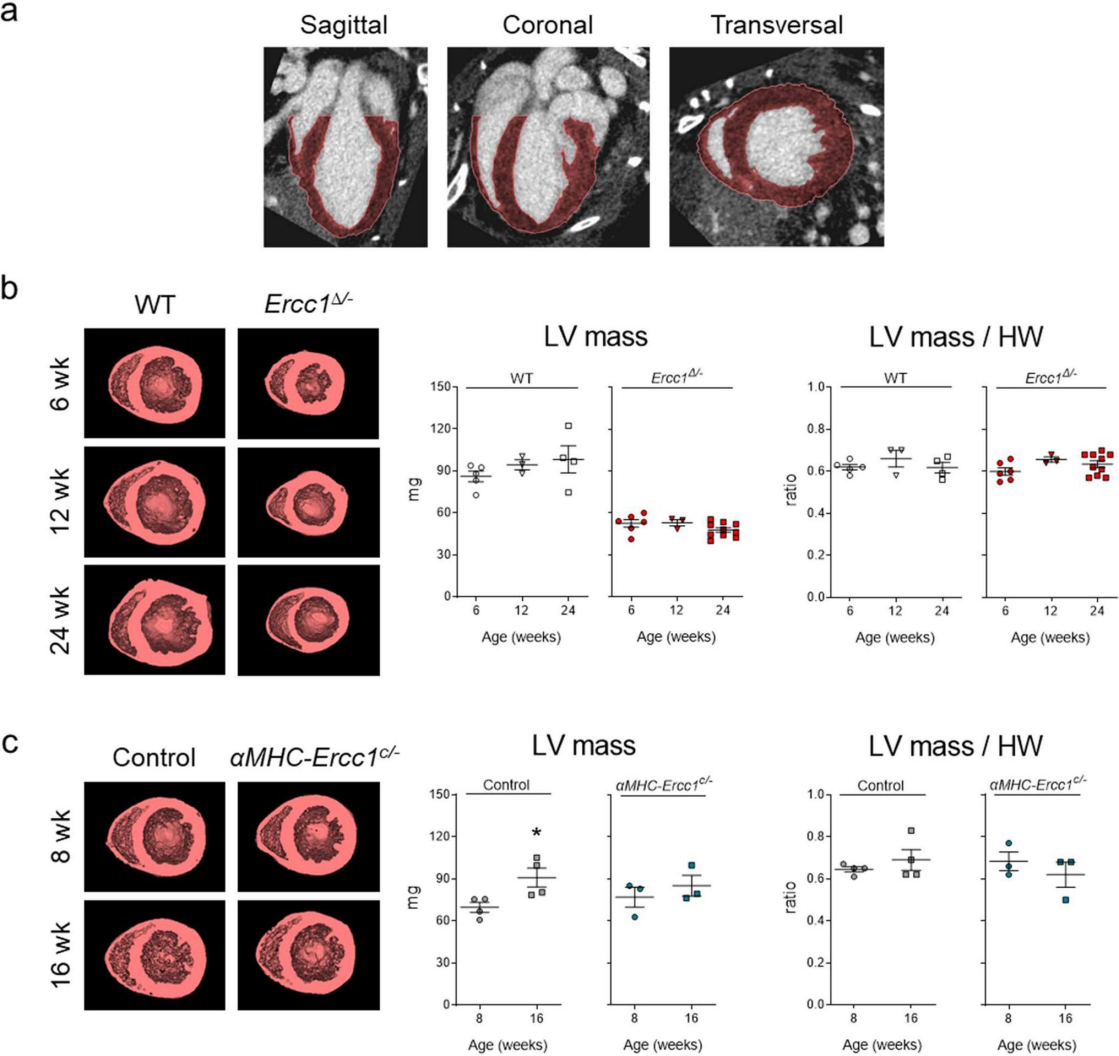
*In vivo* micro-CT derived left ventricular mass in systemic as well as conditional mutants. **a**. Representative micro-CT images of a WT heart in which the myocardial wall is selected (red) for analysis. **b**. Representative 3D reconstructed myocardial wall images of WT and *Ercc1*^*∆/−*^ hearts at 6, 12 and 24 weeks of age and quantification of LV mass and calculation of LV mass to heart weight ratios for all groups. Number of animals per group for 6, 12 and 24 weeks, respectively, *n* = 5, 3, 4 for WT and *n* = 6, 3, 10 for *Ercc1*^*∆/−*^. **c**. Representative 3D reconstructed myocardial wall images of control and *αMHC-Ercc1*^*c/−*^ hearts at 8 and 16 weeks of age and quantification of LV mass and calculation of LV mass to heart weight ratios for all groups. *n* = 4 for both control groups and *n* = 3 for both *αMHC-Ercc1*^*c/−*^ groups. Data is presented as mean ± SEM. Statistical significance *p < 0.05 vs. corresponding 8 weeks

Figure 1a shows representative micro-CT images of a WT heart in which the myocardial wall was selected for analysis. The 3D reconstructed myocardial wall images of WT and *Ercc1*^*∆/−*^ hearts (Fig. 1b) showed a smaller LV mass in *Ercc1*^*∆/−*^ mice compared to WT littermates at 6, 12, and 24 weeks, paralleling the observations on heart weight in Table 1. Consequently, the ratio of LV mass to heart weight was unchanged in both groups at all three ages.

A similar analysis was performed in control and *αMHC-Ercc1*^*c/−*^ hearts at 8 and 16 weeks of age (Fig. 1c). The increases in heart weight in control and *αMHC-Ercc1*^*c/−*^ mice over time as observed in Table 1, was also reflected in increases in LV mass derived from the 3D reconstructed myocardial wall images, resulting in maintained LV mass to heart weight ratios in both groups at both ages (Fig. 1c).

The micro-CT derived LV mass showed an excellent linear relationship with the absolute heart weight, with the LV contributing ~ 62% of the total heart weight in all groups (Suppl. Figure 2).

### Quantitative *In Vivo* Micro-CT Assessment of Left Ventricular Function

**Fig. 2.**
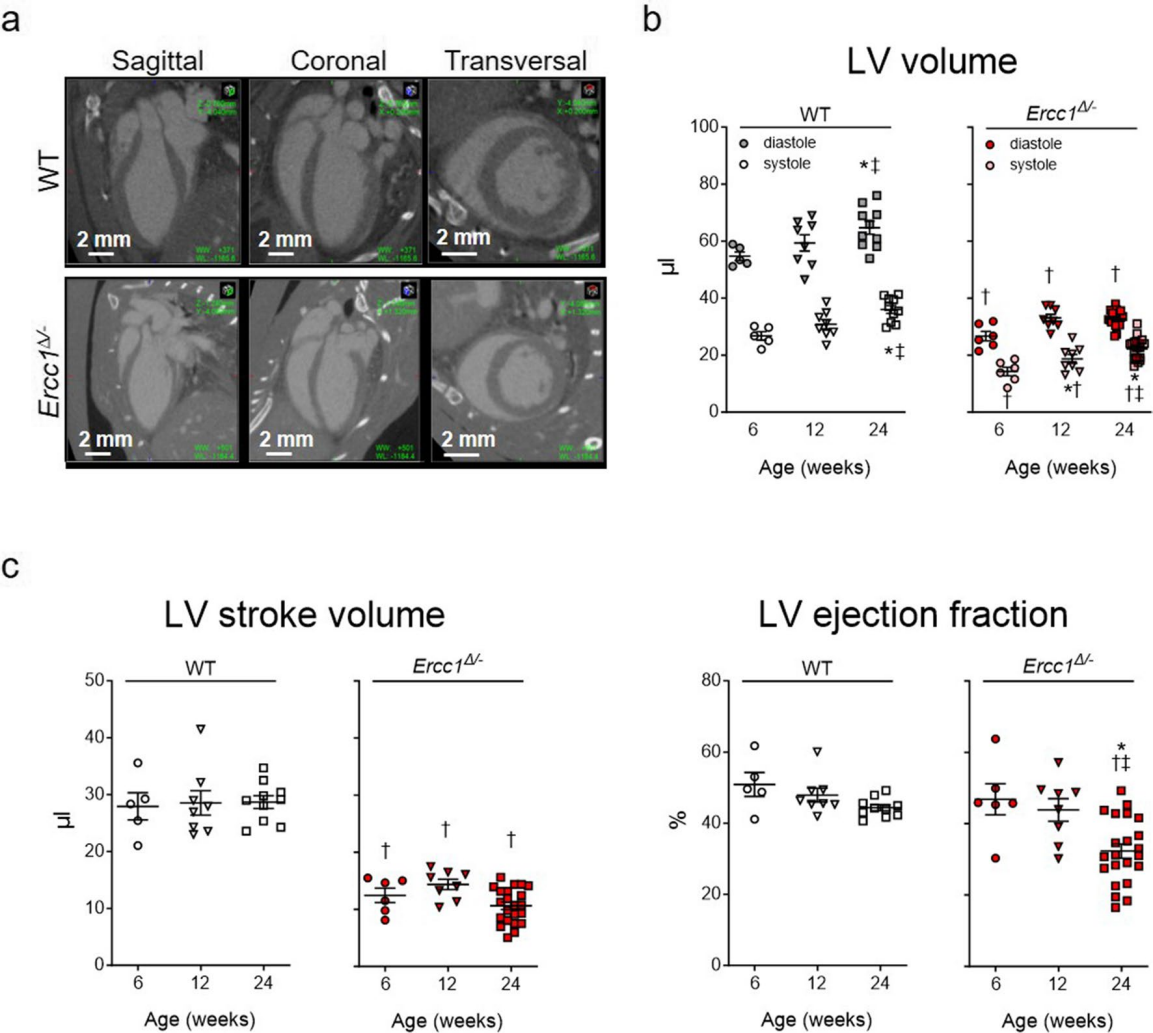
*In vivo* micro-CT derived left ventricular function in *Ercc1*^*Δ/−*^ and WT mice. **a**. Representative micro-CT images of WT and *Ercc1*^*Δ/−*^ hearts in LV end-diastole at 24 weeks of age. **b**. Analysis of LV end-systolic and end-diastolic volumes for all groups. **c.** Calculation of stroke volume and ejection fraction for all groups. Data is presented as mean ± SEM. Number of animals per group for 6, 12 and 24 weeks, respectively, *n* = 5, 8, 10 for WT and *n* = 6, 8, 22 for *Ercc1*^*∆/−*^. Statistical significance *p < 0.05 vs. corresponding 6 weeks; ‡*p* < 0.05 vs. corresponding 12 weeks; †*p* < 0.05 vs. age-matched WT

For each animal, 3D segmentations using a threshold-based approach were conducted on both end-diastolic and end-systolic volumes to measure the LV end-diastolic volume and LV end-systolic volume (Fig. 2a). From these measurements, LV stroke volume and LV ejection fraction were calculated using the formulas outlined in the Methods section and in van Deel et al. [18].The findings of the volume and global functional assessments are presented in Fig. 2. Both diastolic and systolic LV volumes showed an increase over time, but volumes were consistently lower in *Ercc1*^*∆/−*^ mice as compared their WT littermates (Fig. 2b). This was accompanied by a lower stroke volume in *Ercc1*^*∆/−*^ mice compared to WT littermates in all three age groups (Fig. 2c). At 24 weeks of age, ejection fraction (stroke volume normalized to diastolic volume) was significantly decreased in *Ercc1*^*∆/−*^ mice compared to 6 and 12 weeks of age, as well as compared to WT littermates at 24 weeks. These findings indicate a decrease in LV pump function in *Ercc1*^*∆/−*^ mice at advanced age.

**Fig. 3.**
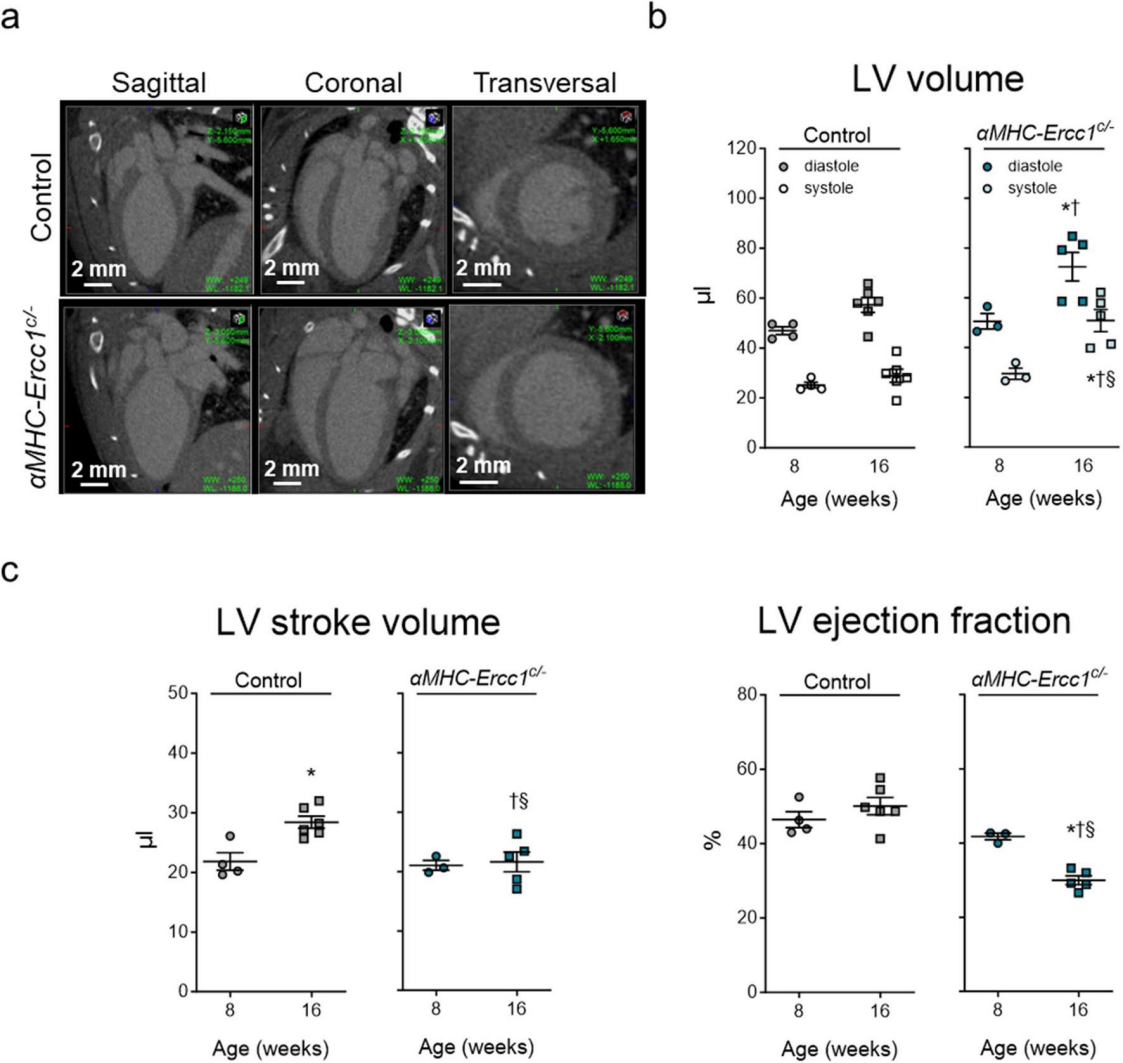
*In vivo* micro-CT derived left ventricular function in *αMHC-Ercc1*^*c/−*^ and control mice. **a**. Representative micro-CT images of control and *αMHC-Ercc1*^*c/−*^ hearts in LV end-diastole at 16 weeks of age. **b**. Analysis of LV end-systolic and end-diastolic volumes for all groups. **c.** Calculation of stroke volume and ejection fraction for all groups. Data is presented as mean ± SEM. Number of animals per group for 8 and 16 weeks, respectively, *n* = 4, 6 for control and *n* = 3, 5 for *αMHC-Ercc1*^*c/−*^. Statistical significance *p < 0.05 vs. corresponding 8 weeks; †*p* < 0.05 vs. age-matched Control; §*p* < 0.05 interaction genotype x age

LV volumes and function were also assessed with micro-CT in 8 and 16-week-old *αMHC-Ercc1*^*c/−*^ mice and control littermates (Fig. 3a). At 8 weeks of age, LV diastolic and systolic volumes were not different between *αMHC-Ercc1*^*c/−*^ mice and control littermates (Fig. 3b). However, at 16 weeks of age, LV diastolic and systolic volumes in *αMHC-Ercc1*^*c/−*^ mice were markedly increased compared to 8 weeks as well as their age-matched control littermates, which resulted in a smaller stroke volume compared to their age-matched control littermates (Fig. 3b, c). Consequently, at 16 weeks, the ejection fraction was significantly decreased compared to their age-matched control littermates and the 8 week time point.

### *In Vivo* and *Ex Vivo* Examination of Myocardial Apoptosis

**Fig. 4.**
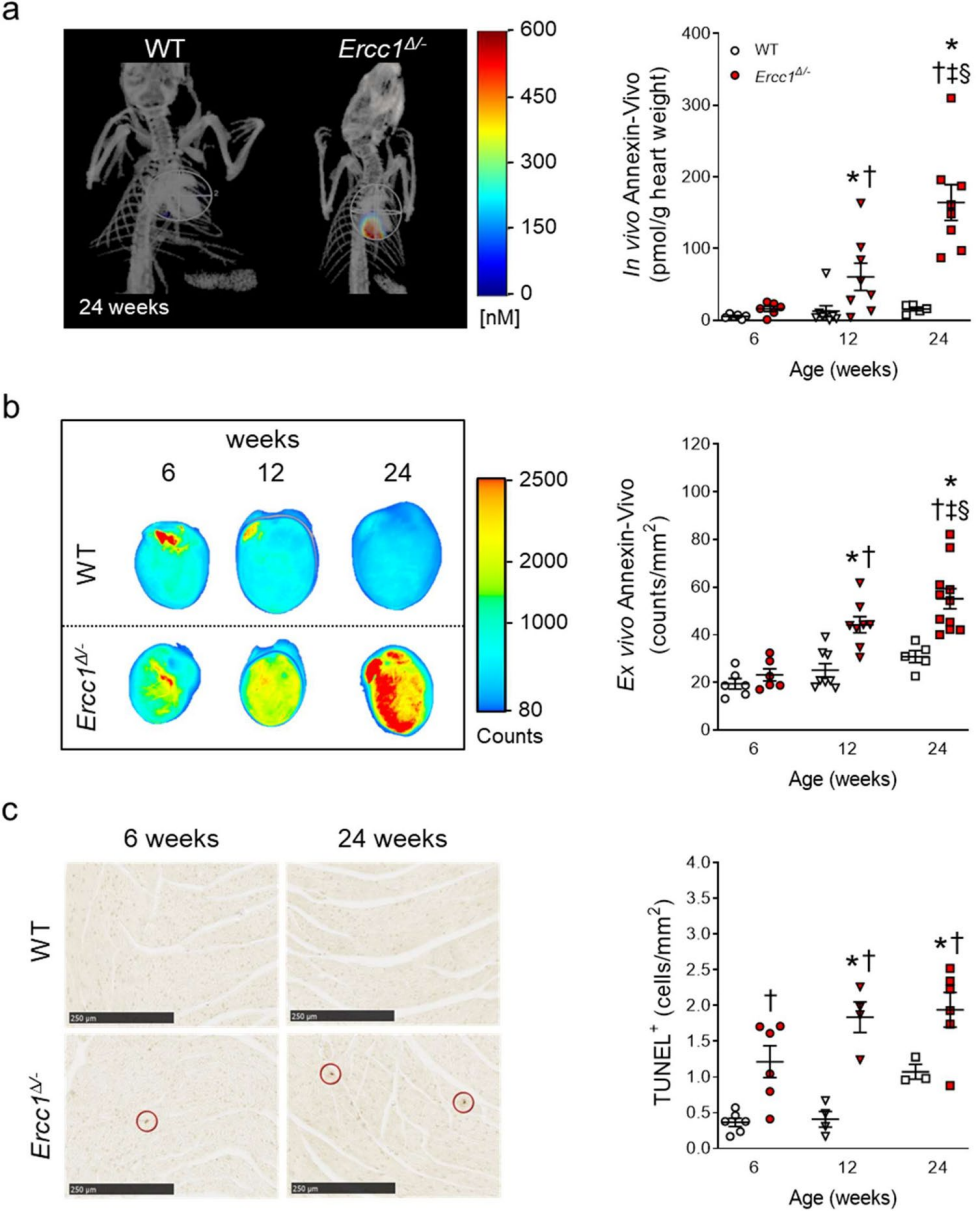
*In vivo* and *ex vivo* examination of myocardial apoptosis in *Ercc1*^*Δ/−*^ and WT mice. **a**. Left: *In vivo* micro-CT-FMT imaging of apoptosis in *Ercc1*^*∆/−*^ mice and WT littermates, showing representative images of mice at 24 weeks of age. Signal from other tissues is excluded for clarity. Right: Quantification of *in vivo* myocardial apoptosis by dual fusion of FMT and micro-CT imaging. Number of animals per group for 6, 12 and 24 weeks, respectively, *n* = 6, 8, 5 for WT and *n* = 6, 8, 8 for *Ercc1*^*∆/−*^. **b**. Left: Representative epifluorescence 2D images of excised hearts obtained with the Odyssey imaging system. Right: Quantification of *ex vivo* myocardial apoptosis. Number of animals per group for 6, 12 and 24 weeks, respectively, *n* = 6, 8, 5 for WT and *n* = 6, 8, 11 for *Ercc1*^*∆/−*^. **c.** Left: Representative images of TUNEL-stained LV sections. Right: Quantification of TUNEL-positive nuclei. Number of animals per group for 6, 12 and 24 weeks, respectively, *n* = 6, 4, 3 for WT and *n* = 6, 4, 6 for *Ercc1*^*∆/−*^. Data is presented as mean ± SEM. Statistical significance *p < 0.05 vs. corresponding 6 weeks; ‡*p* < 0.05 vs. corresponding 12 weeks; †*p* < 0.05 vs. age-matched WT; §*p* < 0.05 interaction genotype x age

To investigate whether we could non-invasively detect apoptotic cells in the aging *Ercc1*^*∆/−*^ hearts, we injected these animals with the NIRF probe Annexin-Vivo. Micro-CT-FMT-reconstructed 3D images showed an increased intensity of Annexin-Vivo in *Ercc1*^*∆/−*^ compared to WT littermates (Fig. 4a). Quantification of the *in vivo* fluorescent signal revealed a consistently low level of apoptosis in aging WT mice, and demonstrated a progressive increase in myocardial apoptosis in *Ercc1*^*∆/−*^ mice. 2D tissue epifluorescence imaging of excised hearts confirmed the significantly increased fluorescence seen non-invasively by FMT in *Ercc1*^*∆/−*^ mice (Fig. 4b). Moreover, both the *in* and *ex vivo* increase in Annexin-Vivo signal correlated significantly with a functional decline in LV ejection fraction (Suppl. Figure 3a).

Immunohistochemistry confirmed the presence of apoptotic cells in the myocardium of 6-, 12- and 24 week-old *Ercc1*^*∆/−*^ mice. Quantification of TUNEL staining revealed a significant increase in TUNEL-positive cells in *Ercc1*^*∆/−*^ hearts compared to WT littermate hearts (Fig. 4c).

**Fig. 5.**
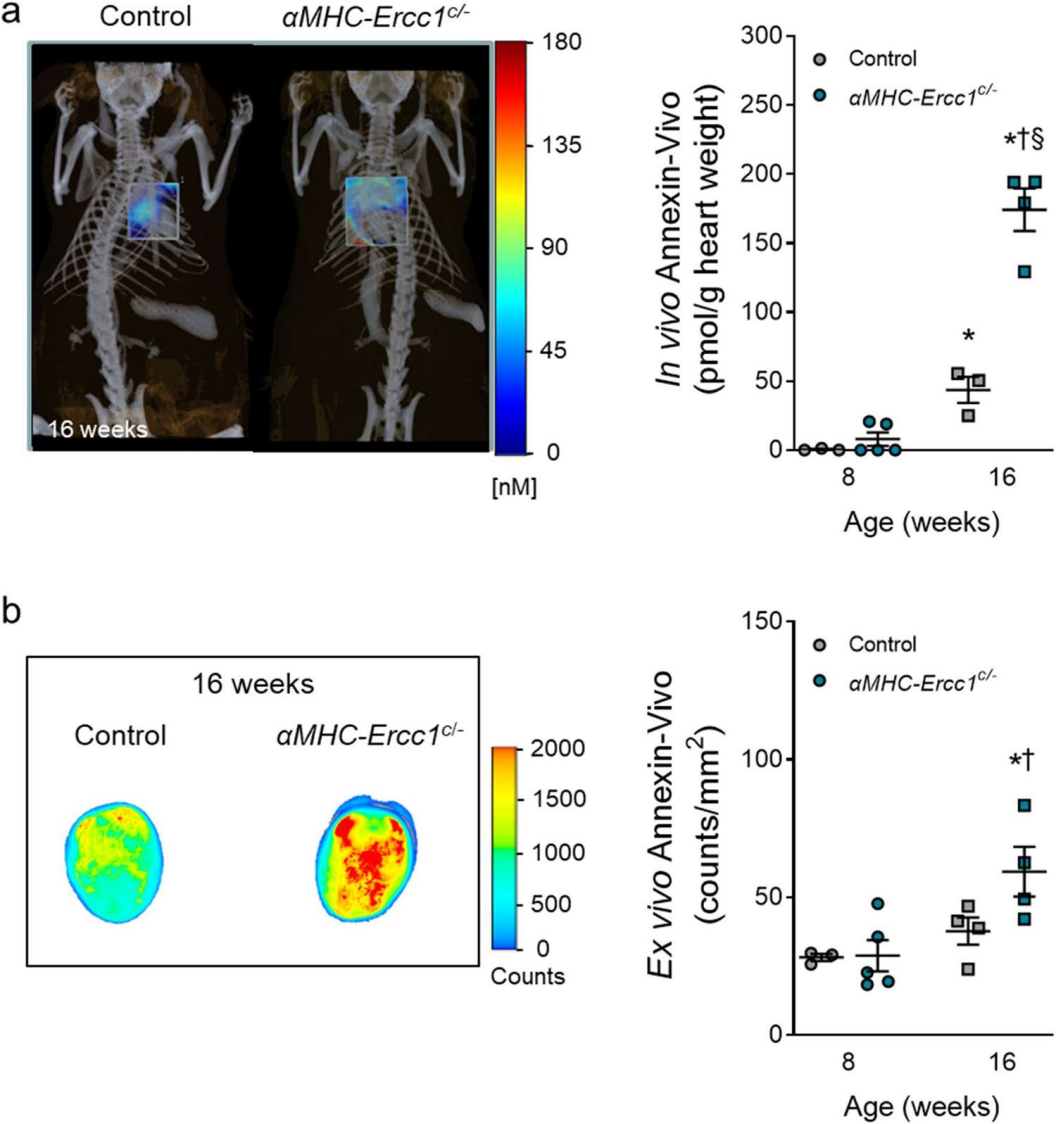
*In vivo* and *ex vivo* examination of myocardial apoptosis in *αMHC-Ercc1*^*c/−*^ and control mice. **a**. Left: *In vivo* micro-CT-FMT imaging of apoptosis in *αMHC-Ercc1*^*c/−*^ mice and control littermates, showing representative images of mice at 16 weeks of age. Signal from other tissues is excluded for clarity. Right: Quantification of *in vivo* myocardial apoptosis by dual fusion of FMT and micro-CT imaging. Number of animals per group for 8 and 16 weeks, respectively, *n* = 3, 3 for control and *n* = 5, 4 for *αMHC-Ercc1*^*c/−*^. **b**. Left: Representative Epifluorescence 2D images of the excised hearts obtained with the Odyssey imaging system. Right: Quantification of *ex vivo* myocardial apoptosis. Number of animals per group for 8 and 16 weeks, respectively, *n* = 3, 4 for control and *n* = 5, 4 for *αMHC-Ercc1*^*c/−*^. Data is presented as mean ± SEM. Statistical significance *p < 0.05 vs. corresponding 8 weeks; †*p* < 0.05 vs. age-matched Control; §*p* < 0.05 interaction genotype x age

To investigate whether specific *Ercc1* deletion in cardiomyocytes also resulted in increased apoptotic activity, we injected 8 and 16-week-old αMHC-*Ercc1*^*c/−*^ and control mice with the Annexin-Vivo probe. At the age of 16 weeks, the Annexin-Vivo signal significantly increased in *αMHC-Ercc1*^*c/−*^ hearts compared to 8 weeks as well as compared to age-matched control littermates *in vivo* (Fig. 5a)*.* 2D tissue epifluorescence imaging of excised hearts confirmed the increased fluorescence in 16-week-old *αMHC-Ercc1*^*c/−*^ hearts (Fig. 5b). In addition, both the *in* and *ex vivo* increase in Annexin-Vivo signal correlated significantly with a functional decline in LV ejection fraction (Suppl. Figure 3b).

## Discussion

In the present study, we explored apoptosis as a potential biomarker for cardiac failure. For this purpose, we utilized functional micro-CT and FMT imaging techniques to assess cardiac function and detect apoptosis in the heart in real-time. The key findings of our investigation are as follows: (i) Consistent with recent observations [14, 15], both systemic and conditional *Ercc1* mutant mice exhibited left ventricular (LV) dysfunction and elevated levels of apoptosis at an advanced age. (ii) Notably, our study revealed that apoptosis could be detected in systemic *Ercc1* mutant mice prior to the development of LV dysfunction, indicating its potential as an early indicator of cardiac impairment. (iii) Interestingly, our results demonstrated that apoptosis could be detected *in vivo* as early as 12 weeks of age, a time when mice still exhibited apparently normal LV function. This suggests that hybrid imaging of apoptosis holds promise as an early marker for detecting LV dysfunction. Overall, our study highlights the utility of combined functional micro-CT and FMT imaging in assessing cardiac function and detecting apoptosis, providing valuable insights into the potential of apoptosis as an early biomarker for cardiac failure.

Hybrid imaging optimized for small animals is very important because of the widespread use of genetically engineered mice recapitulating disease and the need to investigate *in vivo* the functional, anatomical in combination with the molecular phenotype of these mice. The advances in new and existing imaging modalities for small animals allow more accurate, high resolution, 3D, longitudinal imaging of the cardiovascular system and provides rapid translation of new knowledge to the clinic [19]. Micro-CT imaging is frequently used for characterization of cardiac function and structure in small animals, and current systems now provide cardio-respiratory gating, to minimize movement interference and discriminate between systolic and diastolic phase [20, 21]. The present study shows that micro-CT measurements of LV mass corresponded well with post-mortem assessment of heart weight. Moreover, using micro-CT imaging we observed that both systemic and cardiomyocyte-restricted *Ercc1* mutant mice displayed a reduced ejection fraction at the later time point, confirming previous findings with 2D echocardiography [14]. The overall changes in LV geometry and function of the *Ercc1* mutant mice suggest that *Ercc1* deficiency contributes to the development of features consistent with progressive dilated cardiomyopathy (DCM) [22]. DCM is a disease of the myocardium that is characterized by ventricular chamber enlargement with a reduction in cardiac performance. Several studies have already indicated a link between DNA damage and DCM in humans [5] as well as animal models [6, 14, 15].

Fluorescent molecular imaging data presented in this study revealed that LV dysfunction in the systemic *Ercc1* mutant mice was preceded by a progressive increase in apoptosis, suggesting that myocardial cell death contributes to cardiac remodeling. The prognostic relevance of apoptosis measurements was evaluated by examining the correlation between LV function in *Ercc1* mutant mice and Annexin-Vivo molecular imaging data. This analysis showed a correlation, emphasizing a clear link between heightened apoptosis and the observed decline in heart function. DNA damage is a crucial mediator for cells to undergo apoptosis or enter senescence and it was shown that *Ercc1* deficient cells undergo premature cellular senescence as a result of their genome instability [13]. Additionally, apoptosis has been suggested to be responsible for a significant amount of cardiomyocyte death that contributes to the development and progression of heart failure. Indeed, apoptosis has been observed in several cardiac diseases [23–25]. However, whether there are increases in apoptotic cell numbers in failing hearts remains controversial [26]. One of the arguments is that the TUNEL technique, which detects apoptosis by identifying *in situ* DNA nicks, is not solely specific for programmed cell death but might also label cells that undergo DNA repair [27]. The apoptosis probe we used in this study, Annexin-Vivo, binds to phosphatidylserine exposed on early apoptotic cells and does not detect DNA nicks, and therefore holds potential for *in vivo* identification of apoptosis. In the cardiomyocyte-restricted *Ercc1* mutant mice, cardiac dysfunction and apoptosis were both absent at 8 weeks and both present at 16 weeks. In contrast, fluorescent *in vivo* imaging of Annexin-Vivo in the *Ercc1*^Δ/−^ mice, demonstrated that *Ercc1* deficiency leads to increased myocardial apoptosis already starting at 12 weeks of age before overt changes in cardiac performance occurred. TUNEL staining of the heart indeed confirmed significantly increased myocardial apoptosis in *Ercc1*^Δ/−^ mice at each age examined. Accordingly, this probe holds important potential for *in vivo* assessment of apoptosis involved in cardiac remodeling and failure and can provide valuable insights into early disease detection.

In conclusion, this is the first study that combines micro-CT with molecular imaging to show that *Ercc1* deficient mice develop cardiac pathology which starts with an *in vivo* gradual increase in apoptosis, leading to progressive LV dysfunction. The use of DNA repair deficient progeroid mouse mutants as models in this study offers significant advantages. Unlike aged wild-type mice, where age-related cardiac failure is underrepresented due to cancer susceptibility, these DNA repair deficient mutants exhibit accelerated cardiac aging, making them ideal for studying cardiac failure biomarkers. Additionally, their cardio-specific accelerated aging enables the analysis of selective cardiac parameters in a shorter time frame with fewer animals, making this model more convenient, efficient, and cost-effective for our research. The use of micro-CT is a valuable imaging modality to establish cardiac function in small animals as well as explore 3D cardiac geometry. Moreover, combined CT and optical imaging allows simultaneous analysis of molecular and functional changes in mouse models and holds important potential for early disease detection, exploring disease progression and the assessment of therapeutic effects.

### Supplementary Information

Below is the link to the electronic supplementary material.

Supplementary file1 (DOCX 300 KB)

## Data Availability

The data analyzed during the current study are available on reasonable request to the corresponding authors.
