# Supplementary material for: Hybrid Molecular and Functional Micro-CT Imaging Reveals Increased Myocardial Apoptosis Preceding Cardiac Failure in Progeroid Ercc1 Mice
Source: Mol Imaging Biol. 2024 Mar 18;26(4):628–37. doi: 10.1007/s11307-024-01902-4 (PMC11281969; doi:10.1007/s11307-024-01902-4)
Supplement: Supplementary file 1 — Supplementary file1 (DOCX 300 KB) [file 11307_2024_1902_MOESM1_ESM.docx]

**SUPPLEMENTAL FIGURES**


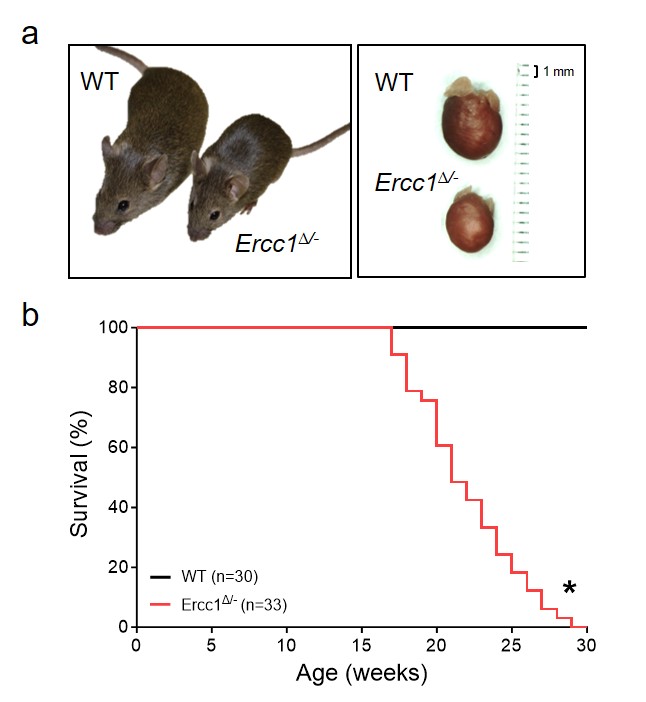


**Supplemental Figure 1. Systemic** ***Ercc1* deficiency results in severe growth retardation and a shortened lifespan.** **a.** Representative pictures of WT and *Ercc1^Δ/-^* mice and hearts at 24 weeks of age. **b.** Lifespan is reduced in *Ercc1^Δ/-^* mice compared to WT littermates. The number of animals is indicated in the graph. Statistical significance *p<0.0001 vs. WT.


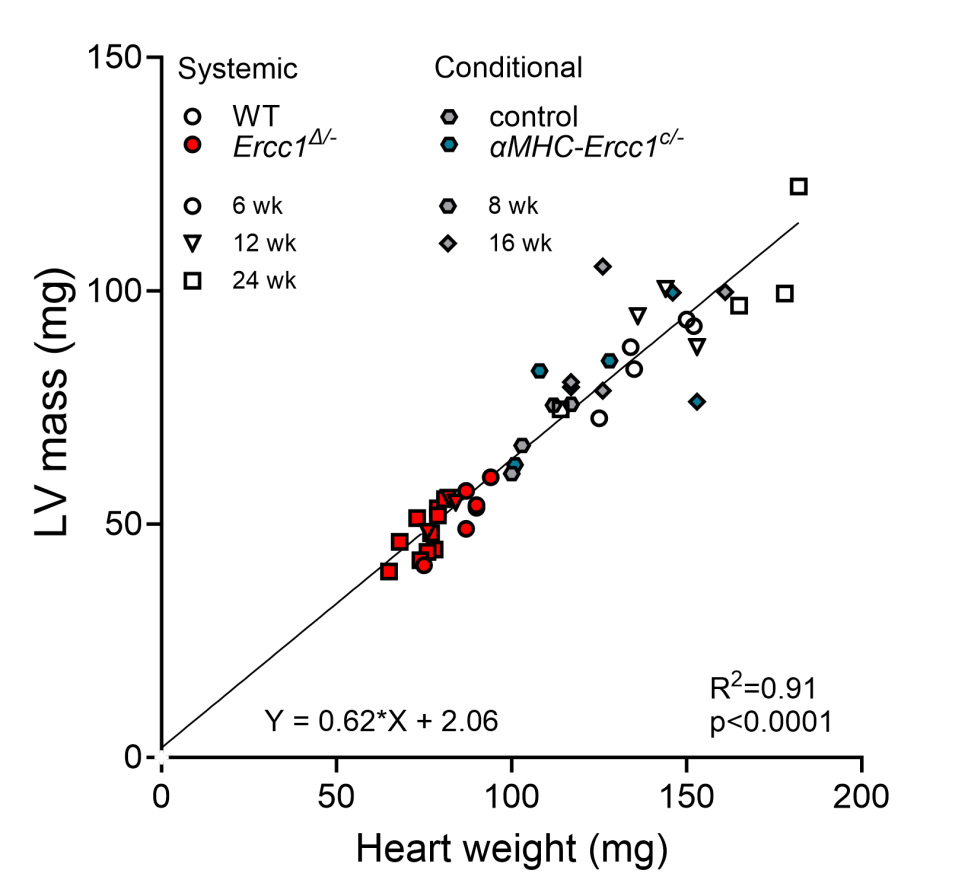


**Supplemental Figure 2. Highly linear and proportional relationship between the micro-CT derived LV mass and the absolute heart weight.** Note that the LV constitutes ~62% of total heart weight.


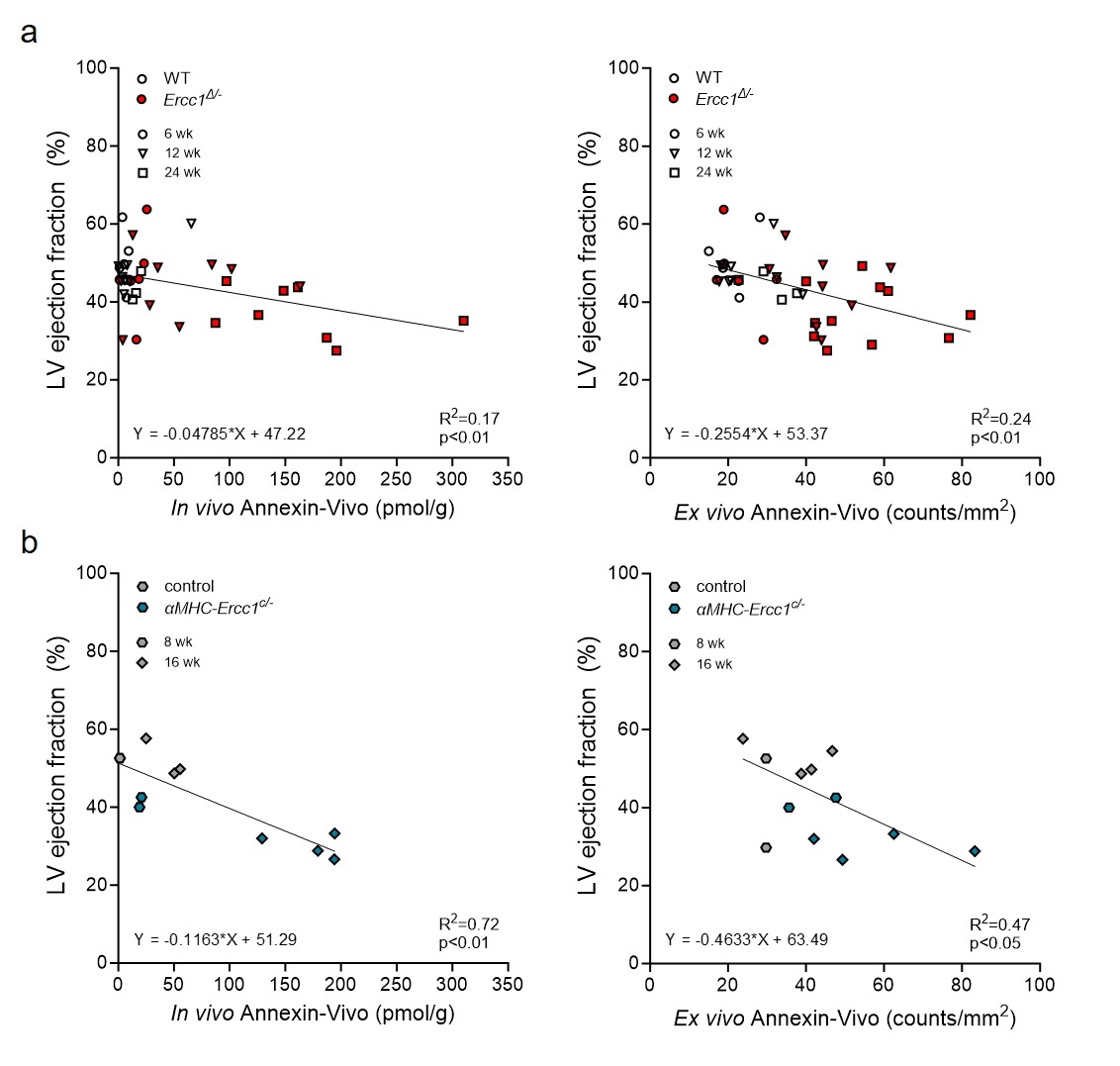


**Supplemental Figure 3. Correlation between cardiac function and myocardial apoptosis. a.** Graphs depicting the micro-CT derived LV ejection fraction on the y-axis and the *in* (left) or *ex* (right) *vivo* Annexin-Vivo levels on the x-axis for *Ercc1^∆/-^* and WT littermates. Number of animals per group for 6, 12 and 24 weeks, respectively, n=5, 8, 4 for WT and n=6, 8, 8-11 for *Ercc1^∆/-^*. **b.** Graphs depicting the micro-CT derived LV ejection fraction on the y-axis and the *in* (left) or *ex* (right) *vivo* Annexin-Vivo levels on the x-axis for *αMHC-Ercc1^c/-^* and control littermates. Number of animals per group for 8 and 16 weeks, respectively, n=1, 3 for control and n=2, 4 for *αMHC-Ercc1^c/-^*.
